# Condensation of Data and Knowledge for Network Traffic Classification: Techniques, Applications, and Open Issues

**DOI:** 10.3390/s25082368

**Published:** 2025-04-08

**Authors:** Changqing Zhao, Ling Xia Liao, Guomin Chen, Han-Chieh Chao

**Affiliations:** 1 School of Electronic Information and Automation, Guilin University of Aerospace Technology, Guilin 541004, China; zhaochq@guat.edu.cn (C.Z.);; 2 School of Management, Guilin University of Aerospace Technology, Guilin 541004, China; 2020152@guat.edu.cn; 3 Department of Applied Informatics, Fo Guang University, Yilan 36247, Taiwan; 4 Department of Artificial Intelligence, Tamkang University, New Taipei City 251301, Taiwan; 5 Department of Electrical Engineering, National Dong Hwa University, Hualien 974301, Taiwan; 6 Institute of Computer Science and Innovation, UCSI University, Kuala Lumpur 56000, Malaysia

**Keywords:** network traffic classification, concept drift, knowledge transferring, model distillation, dataset distillation

## Abstract

The accurate and efficient classification of network traffic, including malicious traffic, is essential for effective network management, cybersecurity, and resource optimization. However, traffic classification methods in modern, complex, and dynamic networks face significant challenges, particularly at the network edge, where resources are limited and issues such as privacy concerns and concept drift arise. Condensation techniques offer a solution by reducing the data size, simplifying complex models, and transferring knowledge from traffic data. This paper explores data and knowledge condensation methods—such as coreset selection, data compression, knowledge distillation, and dataset distillation—within the context of traffic classification tasks. It clarifies the relationship between these techniques and network traffic classification, introducing each method and its typical applications. This paper also outlines potential scenarios for applying each condensation technique, highlighting the associated challenges and open research issues. To the best of our knowledge, this is the first comprehensive summary of condensation techniques specifically tailored for network traffic classification tasks.

## 1. Introduction

Modern networks are highly complex and dynamic, driven by diverse traffic patterns, heterogeneous devices, and the integration of advanced technologies like cloud computing, IoT, and edge computing. Traffic classification, including malicious flow detection, identifies the relationship between network traffic and its source application, protocol, or service group. This process is essential for ensuring quality of service, optimizing resource utilization, and preventing data breaches and service disruptions in modern networks [1].

Current traffic classification and malicious flow detection approaches typically rely on stationary models trained on large datasets captured under specific conditions. However, these large raw traffic datasets face efficiency and scalability challenges when processed centrally in modern networks, which involve multiple communication protocols, unpredictable traffic flows, and the need for seamless interoperability across domains. Stationary models also suffer from low accuracy, instability, and high latency in such dynamic environments. This is due to the continuous nature of modern network traffic, where datasets collected under specific conditions may not capture the hidden patterns and characteristics of traffic in different scenarios or networks. As a result, the issue of concept drift arises, where the changing statistical properties of network traffic over time degrade the performance of static models [2].

Data condensation is crucial for managing the vast volume of raw traffic data, transforming it into manageable summaries that enable efficient, timely decision making. It abstracts complex spatial and temporal dependencies, facilitating edge-based network management [3] to address data privacy and efficiency challenges in resource-constrained environments such as mobile networks [4], IoTs [5], and VANETs [6]. In dynamic networks, where features evolve over time [2], condensation helps balance the need for rapid adaptation with the necessity of minimizing computational and bandwidth demands to sustain performance and user experience. Additionally, condensation supports scalability, ensuring that network management remains efficient as the network expands. Therefore, the ability to distill actionable insights from large, volatile data streams is vital for effective, sustainable, and secure network management in modern environments.

**Figure 1:**
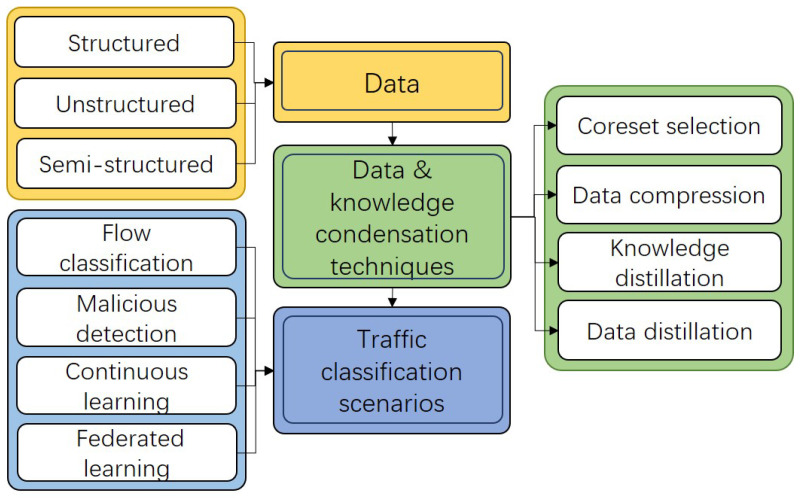
The concepts, methods, and traffic classification scenarios related data condensation introduced in this paper.

This paper reviews key data and knowledge condensation techniques, aiming to establish connections between these methods and emerging application scenarios to address the challenges in current network traffic classification tasks in modern networks. We focus on traditional techniques, such as coreset selection and data compression, alongside newer approaches developed in deep machine learning, including knowledge distillation and dataset distillation. Each technique is categorized from various perspectives, with an introduction to their fundamental concepts, and a summary of typical methods and applications, as illustrated in Figure 1.

We observe that current network (malicious) traffic classification tasks face complex deployment scenarios with severe resource constraints, especially at the network edge. We believe that data and knowledge distillation can significantly improve classification accuracy, resource efficiency, and time efficiency, and the application of data and knowledge distillation in network traffic classification tasks is an emerging area of current and future research. We highlight knowledge distillation and dataset distillation techniques in the context of deep learning, continuous learning, and federated learning, and explore their applications in overcoming challenges such as concept drift, privacy concerns, and resource limitations. We suggest that hybrid approaches that combine two or more types of data and knowledge compression approaches have the best chance for today’s complicated user scenarios and requirements.

To the best of our knowledge, this paper is the first to explore the connection between data condensation techniques and network (malicious) traffic classification, providing a comprehensive review of relevant techniques, algorithms, and applications. Specifically, we present the following key contributions.

- We provide a comprehensive review and classification of data and knowledge condensation techniques and their applications. These techniques are divided into four categories: coreset selection, data compression, knowledge distillation, and dataset distillation. Coreset techniques are categorized by data type, query set, and construction method, while compression techniques are classified by code type, data type, code quality, and coding schemes. Knowledge distillation methods are grouped by distillation schemes, algorithms, and applications, and dataset distillation is categorized based on distillation methods and applications.
- We emphasize data condensation enablers and recent schemes for network (malicious) traffic classification, highlighting the relationship between data condensation techniques and their application in network classification tasks, particularly in complex, resource-constrained environments.
- We examine the gap between advanced condensation approaches and current network (malicious) traffic classification tasks, identifying key challenges and open issues for future research in applying these techniques to traffic classification.

This paper is structured as follows: Section 2 reviews relevant surveys to highlight the novelty and necessity of this study. Section 3 categorizes data into structured, unstructured, and semi-structured types. Section 4 examines four major data condensation techniques—coreset selection, data compression, knowledge distillation, and dataset distillation—summarizing related methods and applications. Knowledge and dataset distillation are emphasized for their high condensation rates, classification accuracy, and data privacy preservation. Section 5 explores the application of these techniques in network (malicious) traffic classification. Section 6 addresses the challenges and open issues in applying condensation methods to traffic classification. Section 7 concludes the paper.

## 2. Related Surveys

Feldman et al. [7] reviewed key methods for constructing coresets, while Jubran et al. [8] focused on the accuracy of coreset methods, and Huang et al. [9] explored coreset selection for time series clustering.

In the area of data compression, Jayasankar et al. [10] conducted a comparative analysis of various compression techniques, evaluating their characteristics, concepts, experimental factors, and limitations, and highlighting open issues for future research. Lungisani [11] reviewed image compression techniques in wireless sensor networks, while Bidwe et al. [12] surveyed deep learning methods in video compression. Chiarot and Silvestri [13] provided a state-of-the-art survey on compression techniques for time series. Additionally, Jia et al. [14] reviewed dimensionality reduction, feature selection, and extraction methods for small datasets, while Tang et al. [15] analyzed compression methods for 3D brain image data, evaluating effectiveness, interpretability, efficiency, and scalability, and identifying open technical challenges for future research in this promising area.

Knowledge distillation, which transfers knowledge between models using teacher–student architectures, has been extensively reviewed. Hu et al. [16] provided a systematic overview of these architectures, learning algorithms, distillation schemes, and their applications in classification, recognition, generation, ranking, and regression. Gou et al. [17] reviewed the distillation from the perspectives of training knowledge, architectures, algorithms, and applications, while Tian et al. [18] focused on distillation for graph-based models, exploring the key aspects of what, who, and how to distill. All reviews noted the challenges and future research directions. Additionally, Alkhulaif et al. [19] examined the distillation techniques in deep learning models, and Liu et al. [20] categorized graph-based distillation methods for deep neural networks, GNNs, and self-knowledge-based approaches, and Luo [21] reviewed distillation techniques for diffusion models. Li et al. [22] focused on applications in computer vision, while Meng et al. [23] explored their use in medical applications.

Dataset distillation, similar to knowledge distillation, transfers knowledge from datasets rather than models. Yu et al. [24] identified 10 key application areas for dataset distillation, including continuous learning, federated learning, neural architecture search, privacy-sensitive applications, security, robustness, graph neural networks, recommendation systems, test classification, and domains like medical, fashion, and design. Lei and Tao [25] reviewed the algorithms for dataset distillation and their performance, while Sachdeva and McAuley [26] classified distillation approaches into meta-model matching, gradient matching, distribution matching, trajectory matching, and factorization-based methods.

Although previous surveys have explored various data condensation techniques, their focus was largely on text and image data. In contrast, our survey targets network traffic flows and aims to provide data condensation strategies for network traffic classification, particularly in edge network deployments. A summary of these surveys is presented in Table 1.

## 3. Types of Data

Data, initially generated by organizations, now include user- and machine-generated data from devices and IoTs. It exists in various forms, such as file systems and relational databases, and is accessed through interfaces like web browsers or query languages. Data are typically categorized as structured, unstructured, or semi-structured [27].

### 3.1. Structured Data

**Figure 2:**
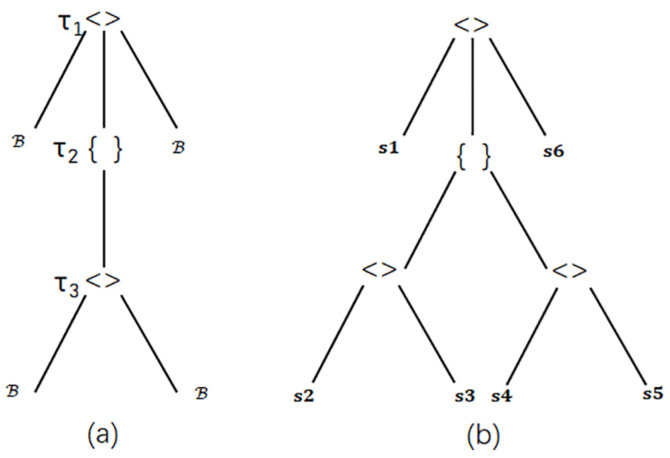
The tree model of structured data. (**a**) The tree representation of schema S1. (**b**) The tree representation of scheme S.

Structured data refer to data with a fixed format and length, highly organized for easy storage and analysis. It is typically stored in relational databases and accessed using SQL with simple search algorithms [28]. Structured data follow a common schema or type, where data values are instances of these schemas. Let $S_{1}=<B,{\left\{<B,B{>}_{\tau 3}\right\}}_{\tau 2},B{>}_{\tau 1}$ be a schema of web pages. It has two tuple constructors (τ1 and τ3) and one set constructors τ2. Let B represent a basic type, and an instance of $S_{1}$ is the value $x_{1}=<t,\left\{<f_{1},l_{1}>,<f_{2},l_{2}>\right\},c>$, where, for instance, *t*, $f_{1}$, and *c* are the book title, the author’s first name, and the cost, respectively. In this context, schemas and values can be viewed as tree structures. Figure 2 illustrates the tree representation of schema $S_{1}$ and value $x_{1}$. A subtree of a schema tree is a subschema, and a sub-value of a value is similarly defined [29].

### 3.2. Unstructured Data

Unstructured data refers to information that lacks a specific structure. Examples include bitmap images, text, customer records, and product lists that are not part of a database [30]. Emails, while stored in a database, are also considered unstructured data due to their text-based, unorganized format. Unstructured data are typically text-heavy but may also include dates, numbers, and facts, making it ambiguous and difficult to process with traditional programs. It is often stored in non-relational databases and accessed via specialized analysis tools, and can be found in formats such as text, image, audio, and video files.

Unstructured data now dominate enterprise data, with estimates suggesting that 70 to 80% of unused organizational data are unstructured [31], growing exponentially from sources like sensors, social media, and digital media [32]. Analyzing these data requires new skills [33].

**Figure 3:**
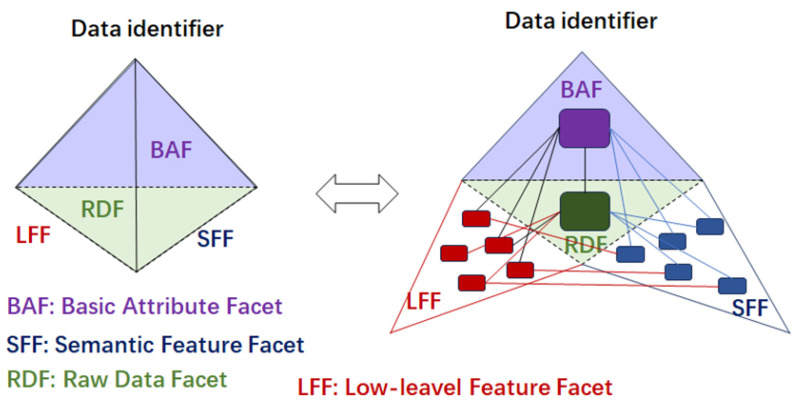
The multilayered model for unstructural data.

As unstructured data are often stored as raw data without a consistent format, it must be described for effective processing. Descriptive unstructured data cannot be represented by relational models [34], but multimedia and spatial data can be modeled using object-oriented (OO) models, which represent data as non-cyclic directed graphs. However, OO models lack a solid theoretical foundation and are complex to implement. Recent research suggests that graphical unstructured data can be represented by logical models [35], and semantic and low-level features can be described using multilayered data models [36] or tetrahedral data models [37]. These models illustrate unstructured data from various perspectives, including basic attributes, semantic features, and raw data, as shown in Figure 3. However, fully describing the complexity of unstructured data remains a significant challenge.

### 3.3. Semi-Structured Data

Semi-structured data refer to data that do not adhere to conventional formatting standards. It is irregular, often incomplete, or subject to rapid or unpredictable structural changes, making it incompatible with fixed schemas [33]. Unlike relational databases or object databases, semi-structured data are not organized in tables or graphs. Typically, it is generated from multiple sources with related but distinct properties that must be integrated, such as emails, XML files, and DOC files [38]. Semi-structured data are often stored as complex objects in object-oriented databases. While keyword searching is commonly used for identification, it often suffers from low efficiency, which can be improved by modeling the data as labeled ordered trees [39].

### 3.4. Summary

**Figure 4:**
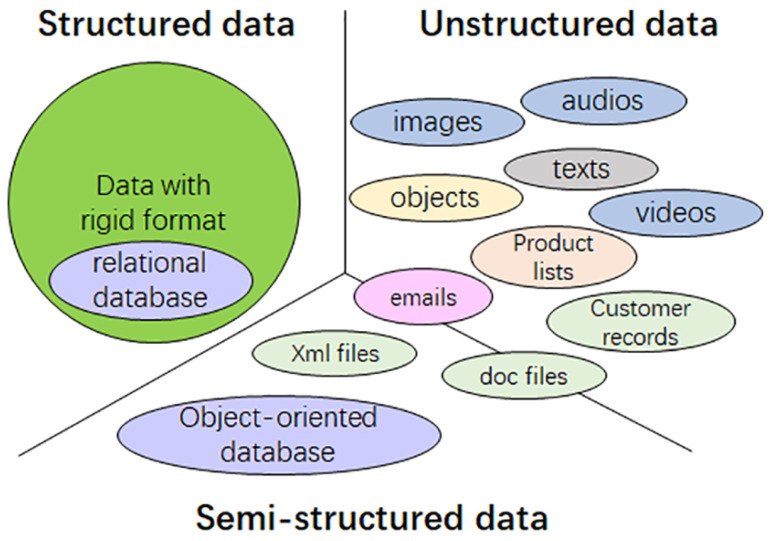
The types of data and their sources.

Figure 4 illustrates the three types of data and their major sources. While the structure of data may not always be explicitly defined, it can be implied in practice. Data may be categorized based on specific tasks, and some structured data may still be treated as unstructured if the structure does not aid in the processing. Similarly, some unstructured or semi-structured data may exhibit hidden or unanticipated structure. Traditional data condensation methods, such as coreset selection and data compression, reduce data size regardless of type. However, techniques like knowledge distillation and dataset distillation are type-sensitive and currently limited to specific unstructured data types, such as images. This review aims to explore data condensation techniques that can facilitate network traffic classification, typically represented as structured data, at the network edge.

## 4. Condensation of Data and Knowledge

Condensation involves summarizing, simplifying, and transforming large volumes of data and knowledge into a smaller, more manageable form. Various techniques can be used for data and knowledge condensation. The remainder of this section discusses coreset selection, data compression, knowledge distillation, and dataset distillation.

### 4.1. Coreset Selection

A coreset is a small subset of data that approximates the full dataset, providing comparable accuracy in estimates [40]. Coresets reduce time, memory, and communication bandwidth by enabling efficient query answering. Solving optimization problems or their approximations on a coreset yields approximate solutions to the original dataset at a significantly lower computational cost. Coresets enhance existing heuristics and bridge the gap between theory and practical systems, improving state-of-the-art methods. They are also useful for processing unbounded streaming data, selecting models, reducing features in machine learning, performing parallel computations across distributed data, minimizing communication bandwidth, enabling real-time computation on devices, and addressing privacy and security concerns [7]. Coresets vary in terms of approximation error and size and can be categorized by data types, query sets, and construction methods, with each category containing several subtypes, as outlined in Table 2.

#### 4.1.1. Data Types

Coresets can be categorized based on the types of data into the following types:

- Weighted subset of input: This is a small weighted subset of the original data, approximating the full dataset by applying loss functions, models, classifiers, and hypotheses on the coreset. It has been used to solve ordered clustering problems [41] and to design efficient algorithms for power mean problems in high-dimensional Euclidean space [42].
- Weighted subset of input space: Here, the input data in both the coreset and the original set are drawn from the same ground set or metric space $R^{d}$. For example, the coreset *S* is a subset of $R^{d}$, but not of the input set *P*. Rosman et al. [43] constructed such a coreset for *k*-segmentation in streaming data, and Tukan et al. [44] used near-convex functions to construct coresets.
- Sketch matrices: In this case, each data point in the coreset is a linear combination of the input data. Feldman et al. [45] used space-efficient sketches of $l_{p}$-distances to approximate $l_{p}$-regression in low-dimensional data streams, while Karnin and Liberty [46] improved streaming sketches for low discrepancy problems.
- Low-dimensional coresets: These coresets represent data in a low-dimensional space rather than using a small number of points. They are often used to reduce the size of sparse datasets [47].
- Generic data structures: These are hybrids of one or more of the above coreset types. Although handling them can be challenging, they may achieve very small coreset sizes for certain problems [43].

#### 4.1.2. Query Set

According to the query set, coresets can be categorized into strong coreset, weak coreset, and sparse solution.

- Strong coresets approximate every query in the given query set, providing error guarantees for all queries [48]. Based on the sensitivity framework introduced by [49] and the sensitivity tight bound of [50], it has been proven that a strong ε-coreset can be obtained through an intelligent reweighting scheme of a normalized weighted input set by selecting a non-uniform random sample of the input.
- Weak coresets are associated with a set of queries but provide error guarantees only for some of them [48]. They can be derived using the Bernstein inequality [51]. Additionally, weak coresets can be obtained from strong coresets by applying non-uniform sampling and reweighting the samples, resulting in smaller coresets compared to those produced by the sensitivity framework [52].
- Sparse coresets provide error guarantees only for the optimal query and are not composable. They typically support specific computational models and do not provide information about the optimal solution of the original data [7]. Sparse coresets can be computed using convex optimization techniques, such as the Frank–Wolfe algorithm [53], or by using the weight vector of the (strong) coreset itself [47].

#### 4.1.3. Constructions

Regarding the construction of coresets, random sampling is the simplest method to reduce dataset size but often fails to preserve the dataset’s structure, especially in noisy real-world data. This is because random sampling disregards information in unselected samples, leading to potential and significant performance degradation [54,55,56]. Current research typically applies uniform sampling, importance sampling, grids, and greedy constructions to construct coresets.

- Uniform sampling divides the input data into subpopulations and samples from each subpopulation in proportion to its size. It is a common method for constructing a kernel and reduces the input time to sublinear time. However, it does not guarantee a (1+ε)-multiplicative error like other coresets and may overlook small or important data points and distant clusters [57].
- Significance sampling constructs a coreset by sampling data from a distribution distinct from the original dataset’s distribution. This distribution is approximated by a weighted average of random draws from the original distribution [58]. It reduces the additive error of the (1±ε)-multiplicative error by replacing a uniform sample with a non-uniform sample of the same size across the input space.
- Grid sampling discretizes the input space into cells and selects a representative from each cell, weighted by the number of input points in the cell. While grid sampling generates kernels with lower additive error, its time and space complexity grows exponentially with the number of cells. Grid-based approaches have been applied to construct coresets for kernel regression [59], dynamic data stream clustering [60], and empirical risk minimization problems [61].
- Greedy constructions generate small-sized coresets for problems with specific properties through an iterative process. In each iteration, the next best point is selected adaptively based on a criterion, such as geodesic alignment [62] or distance minimization [63].

### 4.2. Compression

Based on information theory, data can be highly compressed into fewer bits when its content is already known, resulting in minimal information to be discovered [64]. Data compression, or encoding, involves reducing the number of bytes required to represent data and the memory needed for storage. It consists of an encoding process, where input data is mapped to compressed data, and a decoding process, which reverses the operation to recover the original data. The encoder constructs the coded data, while the decoder performs the inverse operation [65].

Data compression aims to conserve bandwidth in transmission and storage space. For storage applications, encoder requirements are less stringent, as pre-processing can be done offline, but fast and efficient decoding is crucial to minimize response time. Compression techniques can be categorized by codes, data types, quality, and coding schemes, with major strategies summarized in Table 3.

#### 4.2.1. Types of Codes

Compression can be categorized into block codes and variable-length codes. Block codes (also known as block–block codes) refer to compression schemes where the input data and the compressed data have the same length. Common examples include ASCII and EBCDIC, which map data into fixed-length blocks. Although these codes do not provide compression, they are useful for studying transmission rates and capacities.

Variable-length codes, on the other hand, map input data to a variable number of bits, enabling lossless compression and decompression. These can be further categorized into block–variable, variable–block, and variable–variable codes. Variable–variable codes map variable-length input data to variable-length codewords, while block–variable and variable–block codes operate with fixed-length input data and generate variable-length outputs [66]. The primary concern with variable-length codes is how to assemble and parse the data sequence, with some algorithms using predefined word codes and others employing free-parse methods, where the data are divided into variable-length sequences. Well-known variable-length coding strategies include Huffman coding [67], Lempel–Ziv coding [68], and arithmetic coding [69].

#### 4.2.2. Types of Data

Data compression can be categorized into text-based, image-based, audio-based, video-based, and model-based approaches, each aiming to reduce resource consumption in transmission or storage.

Text-based approaches focus on reducing redundancy in text by using predictive models to forecast subsequent characters. These models adapt based on the text processed so far and can be classified into finite-context models and dictionary models. Finite-context models predict the next character based on the fixed context of previous characters, leading to statistical coding methods like arithmetic coding. Dictionary models, on the other hand, replace substrings with references to an evolving dictionary, resulting in dictionary-based algorithms such as Ziv–Lempel coding. For more details, see [70].

Image-based approaches are widely used in applications such as broadcast television, remote sensing (satellite, aircraft, radar, sonar), teleconferencing, computer communications, and facsimile. They are also essential in educational, business, and medical settings for reducing the storage requirements of documents and images. Detailed algorithms for image processing, encoding, and decoding are discussed in [71].

Audio, which encompasses both music and speech, plays a key role in transmission, broadcasting, and storage. Research has identified four audio signal bandwidths for different transmission grades: telephone speech (200–3200 Hz), AM audio (50–7000 Hz), FM audio (50–15,000 Hz), and CD audio (20–20,000 Hz). Audio encoding should be adapted to the specific application for optimal performance. Compression methods are typically evaluated based on quality, bit rate, delay, and complexity, with delay being more critical in two-way voice communication than in one-way broadcasting. Audio compression is generally decoder-intensive, with further details provided in [72].

Video leverages both spatial and temporal techniques, as video can be viewed as a sequence of still images with inherent spatial and temporal redundancy. In this process, the video is first divided into frames, followed by the selection and compression of specific frames. The compressed frames are then reordered to reconstruct the compressed video. The goal is to achieve a low bit-rate content while maintaining high image quality. The methods for selecting and compressing frames vary across different compression approaches. Commonly used video compression techniques include block matching, DCT-based, DWT-based, and hybrid wavelet fractal approaches.

In recent years, deep learning has seen significant advancements in natural language processing, computer vision, and speech analysis. However, these models are often large and require considerable resources. Model compression plays a crucial role in reducing model size, transmission time, bandwidth usage, and computational requirements. Research has demonstrated that a significant amount of redundancy exists among model weights, and a small subset of weights can often reconstruct the entire model [73]. Common model compression techniques include pruning, quantization, knowledge distillation (KD), parameter sharing, tensor decomposition, and subquadratic transformers. Detailed explanations of these methods can be found in [74].

#### 4.2.3. Data Quality

Based on the information loss during compression, methods can be classified into lossy and lossless compression. Lossy compression results in some loss of data, meaning that the original data cannot be fully recovered from the compressed version. In contrast, lossless compression reduces the data size without any loss of significant information, allowing the original data to be perfectly reconstructed.

Lossless compression is commonly used for compressing text files, database tables, and medical images, where data integrity is crucial. Key lossless techniques include run length encoding, arithmetic encoding, Shannon–Fano, Lempel–Ziv–Welch, and Huffman coding. In contrast, lossy compression methods are employed when the original data are not required post-decompression. Lossy methods, such as transform coding, discrete cosine transform, discrete wavelet transform, and fractal compression, are widely used in image, audio, and video compression [75]. Additionally, lossy compression is applied to compress sparse data in scientific computing [76], IoT sensors [77], and deep learning models [78], enabling a significant data size reduction while ensuring that the loss remains either undetectable or acceptable.

#### 4.2.4. Coding Schema

Current research has made significant strides in coding techniques for data compression. This section introduces several widely used methods, including Huffman coding, arithmetic coding, dictionary-based coding, the Burrows–Wheeler transform, scalar and vector quantization, and the wavelet transform. Each of these techniques plays a crucial role in improving compression efficiency across different types of data.

- Huffman coding is an entropy-based algorithm that generates optimal prefix-free codes, commonly used in lossless data compression. It assigns variable-length codes to input characters based on their frequency, ensuring efficient compression by using shorter codes for more frequent characters. Huffman coding operates by constructing a binary tree, where each leaf node corresponds to a character, and the tree’s structure determines the codes. This method is particularly popular due to its simplicity, fast compression speed, and lack of patent restrictions. Huffman coding is employed in various compression schemes, including Deflate, JPEG, and MP3. For further details, see [67].
- Arithmetic coding is an entropy-based technique used in lossless data compression. Unlike traditional methods like Huffman coding, which assigns a fixed-length code to each symbol, arithmetic coding encodes an entire message as a single number, represented as an arbitrary-precision fraction, where 0.0≤qł1.0. This allows frequently occurring characters to be represented with fewer bits, leading to more efficient compression. Arithmetic coding offers greater compression efficiency, supports adaptive models, and computes effectively. It is widely used in adaptive text compression, non-adaptive coding, compression of black-and-white images, and coding of integers with arbitrary distributions [79,80].
- Dictionary-based encoding identifies repeated patterns in an input sequence and encodes them using indices to a dictionary. This method is particularly effective when the input contains frequent patterns, which can be categorized as common or uncommon. Common patterns are encoded with shorter codewords, while uncommon ones use longer codes. The dictionary can be either static or dynamic: a static dictionary is used when prior knowledge of the source is available, whereas a dynamic dictionary adapts to the data when such knowledge is absent. If a pattern is not found in the dictionary, it is encoded using a less efficient method. A well-known dictionary-based technique is the Lempel–Ziv algorithm, which replaces frequently occurring patterns with a single symbol and maintains a dictionary of these patterns. The dictionary’s size is usually fixed. Lempel–Ziv is widely used for lossless file compression, particularly for larger files, and is adaptable to various formats such as GIF, PNG, PDF, and TIFF. Popular versions include LZ77 [81], LZ78 [82], and others [83]. These versions are commonly used in compression utilities like Gzip and ZIP.
- Burrows–Wheeler Transform (BWT) is a lossless block-sorting compression technique (Burrows, 1994). Given an input string *s*, the BWT generates a permutation of *s*, denoted as bwt(s), which allows for efficient compression while still enabling the original string to be retrieved. Compressing bwt(s) is more efficient than directly compressing the original string. Key techniques used in BWT compression include the move-to-front transform [84] and run-length coding [85,86]. Research has demonstrated that BWT achieves high compression ratios with relatively low time and space complexity. The Unix bzip2 utility employs BWT for compression. BWT is particularly useful in biological sciences, where genomes often contain many repeats but few runs. For more details, see [87].
- Fractal compression is a lossy technique used to compress digital images, particularly effective for natural images or textures that exhibit self-similarity within the image. By identifying similar regions in the image and converting them into fractal codes, the image can be compressed efficiently [88]. The technique is based on the iterated function system (IFS), which uses the collage theorem to select transformations that optimize the compression result. Fractal image compression typically involves three steps: image segmentation, similarity search, and similarity coding. The similarity search is crucial, as having sufficient similar regions improves the compression quality but increases computational cost. Several fractal image compression techniques are discussed in [89]. This approach has been widely used in commercial applications for both image and video compression [90].
- Wavelet transform is a data compression technique that converts the input from the time–space domain to the time–frequency domain, achieving more efficient compression. Wavelets are functions defined over a finite interval, and the wavelet transform represents an arbitrary function f(x) as a linear combination of these wavelets or basis functions. These functions are derived from a prototype wavelet, known as the mother wavelet, through scaling and shifting [91]. Wavelet transform is widely used for image compression, with notable implementations including JPEG 2000, DjVu, and ECW for still images, and JPEG XS, CineForm, and the BBC’s Dirac for video. The discrete wavelet transform is also employed in audio compression [92]. The primary goal is to store image or audio data using as little space as possible. Wavelet transform compression can be either lossless or lossy. For more details, see [93].
- Scalar and vector quantization is a lossy compression technique where a scalar value or a vector is chosen from a finite set of possible values or vectors to represent a sample or an input vector of samples, respectively. By reducing the precision of the input data, quantization significantly lowers the computational complexity of compression. This method is commonly used for image compression. In the context of deep learning, large models consisting of weights, biases, and activations can often be quantized to eight-bit integers, making scalar and vector quantization a useful approach for model compression [94].

### 4.3. Knowledge Distillation

Knowledge distillation is a technique developed to address the challenges of deploying large deep neural networks, commonly used in fields like computer vision and natural language processing, on resource-constrained devices such as mobile phones, embedded systems, and autonomous vehicles. While methods like factorization, pruning, and the compression of convolutional filters can reduce redundancy and lower the storage and transmission cost of large models, these approaches still require the models to be decoded into their full size for execution, maintaining the resource constraints. Knowledge distillation solves this issue by transferring the knowledge from a complex, large model (or an ensemble of models) into a smaller, more efficient model, enabling deployment on resource-limited devices without significant loss of performance.

**Figure 5:**
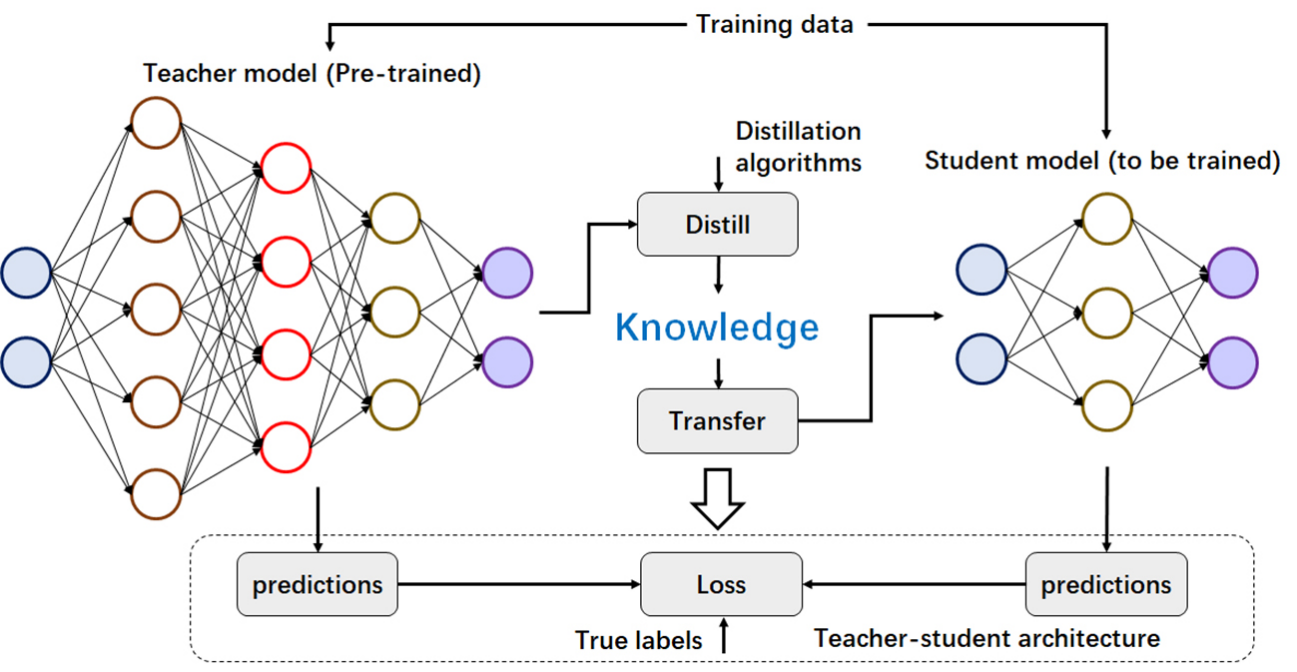
The typical architecture of the knowledge distillation.

Typical knowledge distillation involves transferring knowledge from a large model (or an ensemble of models) to a smaller, more efficient model. This process typically includes three key components: one or more pre-trained teacher models, an initial student model, and a training dataset (as shown in Figure 5). The teacher and student models output class prediction probabilities, and the distillation loss is computed by comparing these predictions. Additionally, the loss between the predicted and true class labels is calculated. Both losses are used to train the student model, optimizing it to achieve an accuracy similar to the teacher model. Knowledge distillation aims to reduce the performance gap between the teacher and student models by efficiently determining their structure. This technique, also known as model distillation, is particularly effective for neural networks with complex architectures and is widely applied in fields such as computer vision, visual question answering, natural language processing, and speech recognition, where large models are impractical due to resource constraints [17].

Knowledge in a neural network model can be categorized into response-based, feature-based, and relation-based knowledge, which correspond to information extracted from the output layer, intermediate layers, and relationships between layers, respectively, [95]. Understanding these different types of knowledge, their core concepts, and how they can be combined is crucial for effective model distillation. In the following, we categorize model distillation approaches based on distillation schemes, algorithms, and applications, as shown in Table 4.

#### 4.3.1. Distillation Schemes

Distillation schemes refer to the methods used to train the teacher and student models in model distillation. Based on these schemes, model distillation can be categorized into offline, online, and self-distillation:

- Offline distillation is the most common approach, where a teacher model is first pre-trained, and knowledge is transferred to train the student model. Due to advancements in deep learning, a wide range of pre-trained neural network models are available as teachers for various use cases. Offline distillation is well established and relatively easy to implement.
- Online distillation addresses the limitation of large teacher models that may not fit into offline scenarios [96]. In online distillation, both the teacher and student models are updated simultaneously during an end-to-end training process [97]. This method is highly efficient and can be operationalized through parallel computing [98].
- Self-distillation involves using the same model as both the teacher and student, making it a special form of online distillation [99]. Unlike traditional online distillation, which requires two steps—first training the teacher model and then transferring knowledge to the student—self-distillation employs a one-step framework that directly trains the student model. This approach typically results in higher accuracy with less training time [100].

#### 4.3.2. Distillation Algorithms

Distillation algorithms refer to the methods used to train student models to acquire knowledge from teacher models. While simple cases can achieve knowledge transfer through the direct matching of response-based, feature-based knowledge, and representation distribution in feature space, more advanced algorithms have been proposed to enhance the knowledge transfer process, particularly in complex settings. Here, we introduce three key distillation algorithms: adversarial distillation, multi-teacher distillation, and cross-model distillation.

- Adversarial distillation refers to distillation algorithms that use generative adversarial networks (GANs) to improve the knowledge transfer between teacher and student models. GANs are machine learning frameworks that generate new data with the same statistical characteristics as the training set [101]. Adversarial distillation can be categorized into three main approaches: (1) using an adversarial generator to create synthetic data, which are then added to the training set [102], (2) employing a discriminator to differentiate between samples generated by the teacher and student models [103], and (3) jointly optimizing both teacher and student models in an online framework [104].
- Multi-teacher distillation involves using multiple teacher models for knowledge transfer during the training of a student network [105]. The simplest approach to transferring knowledge from multiple teachers is to use the averaged response from all teachers as the supervision signal [106]. Additionally, both averaged logits and intermediate layer features can be utilized to enhance dissimilarity between different training samples, further improving the distillation process [107].
- Cross-modal distillation refers to distillation algorithms that transfer knowledge between different modalities, particularly when data or labels for certain modalities are unavailable during training or testing [108]. This approach is commonly applied to distill deep models for tasks such as image, video, and human action recognition.

#### 4.3.3. Distillation Applications

Model distillation has been widely used in visual recognition, natural language processing, and speech recognition, among others.

- Model distillation approaches have found widespread use in visual recognition tasks, such as image/video classification, segmentation, object detection, and estimation. In face recognition, model distillation enhances accuracy for low-resolution images and improves deployment efficiency. By leveraging various types of knowledge from complex data sources, model distillation helps address challenging scenarios and optimize performance across diverse applications.
- In natural language processing (NLP) applications, particularly in neural machine translation [109] and multilingual representation models [110], model distillation is employed to create lightweight, efficient, and effective models, as typical language models tend to be large and resource-intensive. For question-answering systems, distillation improves the efficiency and robustness of machine reading comprehension. By combining sequence knowledge, model distillation effectively transfers information from large networks to smaller, more efficient ones.
- Model distillation is often used to improve the performance of acoustic deep neural models in real-time speech recognition systems deployed on embedded platforms [111]. Model distillation is also used to train deep neural networks to identify acoustic scenes for audio segments [112], or classify environment sound [113].
- Other applications, such as deep recommendation systems [114] and systems that protect deep models from adversarial attacks or perturbations [115], can also take advantage of model distillation for better performance and efficiency.

### 4.4. Dataset Distillation

Instead of transferring knowledge between models, it is also possible to transfer it directly between datasets. This involves distilling the knowledge from a large dataset into a smaller synthetic dataset, enabling a model trained on the smaller dataset to perform comparably to the one trained on the larger dataset. This process is known as dataset distillation. The compact dataset can be transmitted and stored on resource-constrained devices, allowing for the real-time training of small models to perform tasks that would typically require large models and extensive datasets.

The concept of dataset distillation was formally introduced by Wang et al. [116], aiming to extract knowledge from large datasets and generate a significantly smaller synthetic dataset. After providing a brief overview of dataset distillation, the remainder of this subsection outlines its formulation, methods, and applications, as summarized in Table 5.

#### 4.4.1. Formulation and Overview

Given a real dataset T comprising $\left(X_{t},Y_{t}\right)$ and a synthetic dataset S comprising $\left(X_{s},Y_{s}\right)$, $X_{t}$ and $X_{s}$ are of dimensions $R^{|T|\times D}$ and $R^{|S|\times D}$, respectively, where *D* represents the number of features. Similarly, $Y_{t}$ and $Y_{s}$ are of dimensions $R^{|T|\times C}$ and $R^{|S|\times C}$, respectively, where *C* represents the number of classes. The optimization problem formulates the dataset distillation to determine the set of S. This set should minimize the objective of L over both S and T, as demonstrated in Equation (1). Elaborate on the distillation objective L based on the specific distillation tasks.

(1) $$S=arg\underset{S}{min}L\left(S,T\right)$$

**Figure 6:**
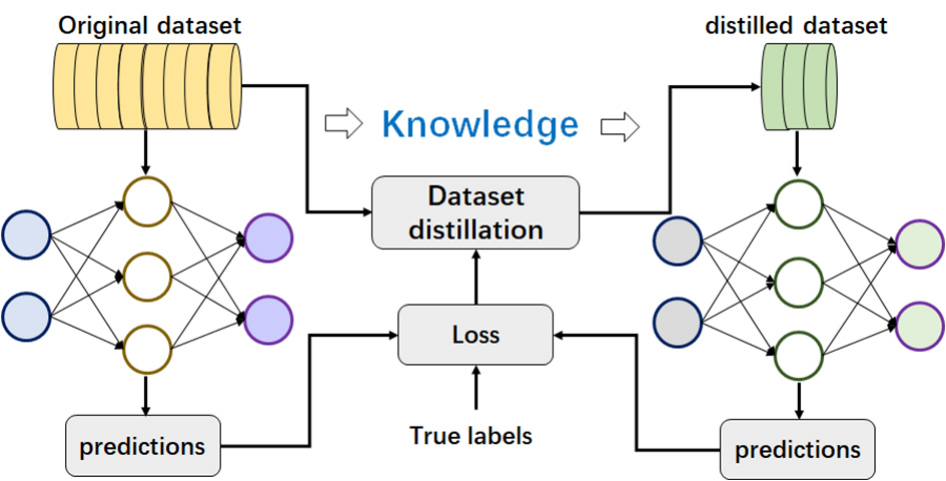
The classical architecture of dataset distillation.

As illustrated in Figure 6, dataset distillation typically utilizes neural networks to condense image datasets. Two essential steps in the distillation process involve training neural networks and iteratively optimizing synthetic datasets S based on the L values computed over these networks.

Specifically, S is first initialized in two ways: through random initialization and by randomly selecting real samples from the original dataset T. Next, a loop optimizes the synthetic dataset by alternately updating the neural network θ and the synthetic data S. Finally, the synthetic dataset is updated by optimizing the distillation objective L. To enhance the convergence and final performance of distillation methods, as well as mitigate overfitting issues and promote generalization of the synthetic dataset, it is recommended to initialize S at the outset. θ can be initialized randomly or loaded from cached checkpoints at the beginning of the loop, and subsequently modified using gradient descent. In some works, the optimization objectives L and the updating sequence of the network and the synthetic dataset may vary.

#### 4.4.2. Methods

Since dataset distillation can be framed as an optimization problem, the approaches employed can be classified into three categories based on their distinct optimization goals: performance matching, parameter matching, and distribution matching.

- Performance matching in dataset distillation, as proposed by Wang et al. [116], is a meta-learning-based approach that optimizes a synthetic dataset to match the performance of models trained on both the synthetic and original datasets. This method uses a bi-level algorithm: in the inner loop, the weights of a differentiable model are updated alongside the synthetic dataset using gradient descent. In the outer loop, models trained in the inner loop are validated on both the synthetic and original datasets, and the performance loss is computed for backpropagation.
- Parameter matching in dataset distillation involves training a neural network on both the original and synthetic datasets, with the goal of ensuring consistency in the network’s parameters. This can be achieved through gradient matching in a single step [117]. However, since the synthetic dataset is updated over multiple steps while the parameters are matched in one step, errors may accumulate. To address this issue, a multi-step parameter matching approach has been proposed [118], where the models trained on both the original and synthetic datasets are optimized to minimize the distance between their parameters.
- Distribution matching aims to generate synthetic data that closely approximate the distribution of real data. This is achieved by minimizing the distance between the two distributions, typically using metrics such as maximum mean discrepancy [119]. However, directly estimating the real data distribution can be computationally expensive and imprecise, especially for high-dimensional data-like images. To overcome this, the maximum mean discrepancy can be approximated using embedding functions [120].

#### 4.4.3. Applications

Dataset distillation has been utilized in numerous applications [24]. Here, we discuss continual learning, federated learning, privacy and security, and others.

- Continual learning refers to the ability of a model to sequentially tackle multiple tasks while retaining knowledge from prior tasks, even when old data are no longer accessible [121]. A primary challenge is catastrophic forgetting, where models tend to forget previously learned information when learning new tasks. To address this, it is essential to preserve prior knowledge in a limited memory buffer. Dataset distillation provides a solution by generating a condensed synthetic dataset that retains the knowledge of the original, larger dataset. Recent dataset distillation approaches [117,119,122] have been applied to scenarios involving continual learning, aiding in the retention of valuable information across tasks.
- Federated learning is a decentralized approach where multiple client devices collaborate with a central global server, allowing each client to train a local model on its data and contribute to a global model, without sharing the local data. Its primary goal is to improve the model performance while maintaining data privacy. However, frequent exchanges of models between clients and the global server can incur high costs. Dataset distillation offers a solution by enabling the exchange of distilled datasets, rather than models, thereby reducing the communication overhead while preserving data privacy [123,124,125,126].
- Privacy and security. Machine learning models are vulnerable to various privacy attacks, including membership inference, model reconstruction, property inference, and model extraction. Dataset distillation addresses these concerns by working with synthetic data, which preserves the privacy of the original dataset. This approach allows for machine learning tasks without exposing sensitive information. Notable works in this area are presented in [20,127,128].
- Others. Dataset distillation has applications in recommendation systems [129] and text classification [130], where models typically require large datasets. By significantly reducing the size of the training data, this method lowers the computational load and improves the time efficiency. Additionally, in medical systems, dataset distillation can generate synthetic data to replace the original data, ensuring the protection of patient privacy [131].

## 5. Condensation in Flow Classification

In this section, we first describe traffic using metadata and provide an overview of flow models. We then review flow classification applications and propose the integration of condensation techniques to enhance their effectiveness.

### 5.1. Metadata of Flows

Flows can be described using metadata, which refers to data that provide information about other data. Metadata can be categorized into three types: content, structural, and context. Content metadata refers to the payloads of packets, which carry the actual information and are typically private, making them less accessible for flow modeling. Structural metadata indicate the relationships between packets in a flow and can be derived by analyzing packet sequences. It is widely used in flow modeling. Context metadata provides external information about the flow, such as who, how, where, and when the flow was generated. This type of metadata can be extracted from the first packet of a flow or from network background data, and it is increasingly utilized for early flow classification. Structural metadata can be further divided into flow-level and packet-level metadata. Flow-level metadata are calculated after a sufficient number of packets have been received, while packet-level metadata are extracted during the early stages of a flow [132].

### 5.2. Flow Models

Flow models can be trained using threshold-based or machine learning (ML)-based approaches. Threshold-based methods classify flows based on whether a particular feature exceeds a predefined threshold, typically applied to flow-level structural metadata, such as byte count. While these models are simple, they often overlook many flow features. In contrast, ML algorithms utilize a broader range of flow features to uncover complex patterns, enhancing model accuracy, efficiency, and stability. As a result, ML-based approaches are increasingly prevalent in current research [133,134].

### 5.3. Traffic Classification Application

- Elephant flow classification applications. Elephant flow classification plays a critical role in data center management. Network traffic is typically divided into elephant and mouse flows, based on their lifetime characteristics. Elephant flows, with long lifetimes, represent a small percentage (10–20%) of total flows but contribute significantly (80–90%) to the overall traffic volume. In contrast, mouse flows, with short lifetimes, make up the majority of flows but only a minor portion of the traffic volume. Classifying flows into these two categories helps inform network management strategies that optimize load balancing, improve link utilization, and mitigate congestion. Elephant flow classification is an essential tool in network traffic engineering, which aims to enhance operational performance, reduce congestion, and control costs. Our prior research has explored various techniques for categorizing elephant flows [132].
- Malicious flow detection. Malicious flows refer to harmful network traffic that can degrade network performance, disrupt normal operations, and compromise devices or servers. Malicious flow detection systems continuously monitor network traffic for signs of suspicious links, files, or connections, and assess whether these links, such as those from blacklisted URLs or command-and-control (C2) sites, constitute malicious activity. These systems leverage large volumes of security data gathered from millions of devices worldwide to identify both known and unknown threats [135]. Due to the encryption of modern Internet traffic, including TCP/UDP port numbers and restricted access to packet payloads, malicious flow detection often relies on machine learning techniques. These methods help train classifiers to identify malicious flows and classify unknown network traffic. For further details on machine learning in malicious flow detection, see [136].
- Continual (incremental) flow classification. Network traffic packets continuously flow through a network, with flows representing sequences of packets that constantly arrive and depart. To accurately classify each flow, a flow classifier must operate on incoming packets in a continual manner. Many traditional flow classification systems assume network traffic characteristics remain stable over time and space, leading to static flow models and classifiers. However, these static models are ill-suited for dynamic environments, where network traffic evolves and flow characteristics vary. The continual (or incremental) learning in flow classification allows for dynamic classifiers that adapt to the changing nature of network traffic. By incorporating continual learning, flow models can be updated to better handle the fluctuations in real-world network traffic, improving detection accuracy and time efficiency, especially for malicious flows [137]. Additionally, continual learning enables models to adapt by updating malicious flow information and improving detection over time [138].
- Federated flow classification. Federated flow classification combines federated learning with network flow classification to create a distributed classifier across multiple clients in a multi-level system [139]. This approach is widely used in mobile networks [4] and the IoT [5] for classifying network or malicious flows, leveraging resource-constrained devices while utilizing edge computing. Federated flow classification allows local traffic to be classified on individual devices, preserving traffic privacy. A global classifier, trained by a central server, improves the performance by integrating insights from multiple local classifiers, without the need to exchange traffic data. This method effectively addresses the challenges related to traffic drift, data privacy, and resource and time efficiency.

### 5.4. Condensation in Network Traffic Classification

Condensation techniques aim to retain the essential information of a primary dataset while reducing its size. These methods are particularly useful in scenarios with limited resources for data storage, transmission, and processing.

- Applying coreset techniques has facilitated incremental learning for mobile network traffic classification [137] and malicious flow detection across various network scenarios. For example, ref. [140] applied Bayesian coresets in Hilbert space to handle large, redundant datasets in network intrusion detection. This approach used a weighted data subset to maintain both efficiency and accuracy. Similarly, ref. [6] employed coreset techniques to extract relevant information from anomalous data, filtering out unnecessary samples, and developed a faster clustering model that minimized the response time and enhanced the performance of intrusion detection in vehicular ad hoc networks.
- Applying data compression in network flow classification aims to reduce redundancy, thereby minimizing memory and bandwidth usage required for the storage and transmission of traffic data [141]. For instance, ref. [142] utilized differential and Huffman coding to compress data for IoT nodes in wireless sensor networks, conserving energy, memory, and bandwidth. Research has also extended data compression to encrypted and compressed datasets. In [143], an approach named high entropy distinguisher (HEDGE) i proposed that trained models on compressed packets for real-time network traffic classification with comparable accuracy. Similarly, ref. [144] introduced a raw data and engineered features classification (RDEFC) approach, which learned patterns from both statistical tests and raw file fragments, enhancing classification accuracy on encrypted and compressed traffic in wireless networks. In federated learning for network traffic classification, compression techniques are employed to compress local and global models, reducing communication costs. Three compression algorithms are evaluated in [145] for federated learning in wireless traffic classification, as detailed in Table 6.
- Applying model distillation has been effective not only in deep learning for neural networks but also in constructing multimodal models for encrypted network traffic classification, achieving high accuracy with minimal memory and time cost [146]. It has also been used to address catastrophic forgetting in malicious traffic classification through incremental learning [138], and to implement in-network intelligence using lightweight student models, which significantly reduce the model size and training time on network devices [147]. Additionally, ref. [148] proposed lightweight deep learning models with up to 50% and 30% reductions in floating-point operations per second (FLOP) and parameters, respectively, for traffic classification on IoT devices.
- Applying dataset distillation has primarily been used in neural networks for image classification but has not yet been directly applied to network flow or malicious flow classification. However, since network traffic flows can be transformed into virtualized images [149,150], neural network-based dataset distillation could potentially be adapted to distill flow traffic datasets. While [151] proposed a Bayesian multilayer perception (BMLP) method, which reduces input data bytes by distilling important bytes for accurate network traffic classification with low memory and time cost. BMLP differs significantly from the typical dataset distillation approaches discussed in this paper.

### 5.5. Comparison of Condensation Methods in Network Traffic Classification

This subsection compares four condensation methods applied to network traffic classification. For each method, we summarize its purpose and discuss its impact on performance.

Coreset selection and data compression are classic techniques used to reduce data size, making them essential for resource-constrained environments. Coreset selection is an efficient method that eliminates redundant data, preserving performance while reducing dataset size. In network traffic classification, it is particularly effective for tasks like malicious traffic detection [140]. In cases with limited resources, such as incremental learning for mobile networks or intrusion detection in VANETs, coreset selection helps reduce the training dataset size, improving the response time without sacrificing accuracy. Data compression, whether lossy or lossless, further minimizes storage and bandwidth requirements. Recent studies have used compressed and encrypted traffic data for training, improving resource efficiency and enhancing privacy protection, as shown in Table 6. These techniques optimize performance and address the constraints of resource-limited devices in dynamic network environments.

**Table 6:** Performance comparison of typical applications, IL—incremental learning, SR—size reduction, TR–time reduction, AR—accuracy reduction.

Methods	Ref.	Type	Purpose	SR	TR	AR
Coreset	[140]	Weighted subset of input	Redundancy reduction	98%	10–50%	10–30%
	[137]	Herding section	Size reduction for IL	44%	90%	5.4%
	[6]	Weighted subset of input	Size reduction in VANET	-	79+%	5+%
Data compression	[142]	Differential/Huffman coding	Memory/bandwidth saving	85%	-	0
	[143]	ZIP/RARBZIP2/GZIP	Size reduction	91%	-	8%
	[144]	BZIP2/ZIP/RAR/GZIP	Size reduction	-	-	10%
	[145]	PQ/QSGD/TopK	Bandwidth saving	74+%	74+%	8%
Model distillation	[146]	Parameter matching	Multimodal learning	82%	56%	7%
	[138]	EWC/GEM	Info. forgetting in IL	-	-	+80%
	[147]	Distribution matching	Model reduction	80%	99%	+3%
	[148]	Two-step distillation	Model compression	30%	50%	0.37%
Dataset distillation	[151]	Byte importance distillation	Size reduction	132	234	37%
	[149]	-	Traffic image conversion	-	-	-
	[150]	-	Traffic image conversion	-	-	-

Model distillation and dataset distillation are advanced data condensation techniques that facilitate the transfer of knowledge from large models or datasets to smaller, more efficient ones, making them ideal for resource-constrained devices in IoT networks, VANETs, and wireless networks. Recent research has introduced several model distillation frameworks for network traffic classification, employing techniques such as parameter matching, distribution matching, and performance matching. These methods effectively reduce model size and computation time while maintaining accuracy or even achieving improvements. While dataset distillation is commonly applied in image classification, its use in network traffic classification remains limited due to the tabular nature of traffic data. However, since network traffic can be converted into virtualized images, existing dataset distillation methods could be adapted for traffic classification. Additionally, new approaches tailored to non-image classification tasks are expected to emerge, offering further potential to enhance network traffic classification efficiency in resource-constrained environments.

## 6. Challenges and Open Issues

This section discusses the open research issues, limitations, and opportunities in the area of network traffic classification for the future research.

- Dealing with the class imbalance in network traffic:
  Class imbalance is a significant challenge in machine learning (ML), particularly in network traffic classification, where unequal class distributions can severely affect model performance. This issue arises when the majority classes dominate, leading to biased models that perform poorly on minority classes. In network traffic, this imbalance is especially pronounced due to the inherent characteristics of Internet traffic. For instance, malicious traffic is often rare compared to benign traffic. Additionally, in network flow analysis, 90% of the flows are "mouse flows," which are long-lived and consume most of the bandwidth, while only 10% are "elephant flows," which are short-lived and consume little bandwidth.
  To address class imbalance, two common solutions include random oversampling and synthetic minority oversampling. These techniques aim to balance class distributions by augmenting the minority class with additional samples. However, network traffic classification poses unique challenges for such techniques due to the complexity of network data [152]. Neural networks, in particular, tend to over-classify majority classes due to their increased prior probability [153], which can significantly degrade the performance of models—especially teacher models—in classifying minority classes. This, in turn, exacerbates the difficulty of knowledge distillation, where the accuracy of student models on minority classes is further compromised.
  To mitigate this, incorporating additional relational information, such as soft labels or pairwise similarities between data points across different classes [154], or combining knowledge distillation with reinforcement learning techniques [155], may help improve the learning of minority classes. For dataset distillation, the challenge becomes even more pronounced, as generating a synthetic dataset that maintains the accuracy for minority classes comparable to the original is difficult. However, this specific aspect has not yet been adequately addressed in the literature, and further research is needed to develop effective strategies for class imbalance in dataset distillation.
- Applying knowledge distillation on non-neural network-based models: Neural networks are large, resource-intensive models, and training them demands significant time and computational power. As such, transferring knowledge from neural networks to non-neural models—or between non-neural models—becomes crucial for extending knowledge distillation to network traffic classification tasks. This also helps improve the efficiency of such tasks, particularly in resource-constrained environments like the network edge. A deep understanding of the types of knowledge present in neural networks and how to effectively combine these different knowledge types is central to the success of knowledge distillation.
  Non-neural network-based models are commonly used in flow classification and other classification tasks, so developing methods for transferring knowledge to and from these models presents a challenge. One possible solution is to convert non-neural models into neural networks, thereby leveraging existing knowledge distillation techniques. Some researchers suggest using decision tree-based models for knowledge distillation, as decision trees can be derived from or mapped to neural networks [156,157]. However, this approach may face significant challenges in enhancing resource efficiency, given the inherent resource demands of neural networks.
- Using dataset distillation in non-image classification tasks: The application of dataset distillation in non-image classification tasks is an emerging area of research. While dataset distillation is well established in neural networks for image classification, its effectiveness in non-image tasks, such as network traffic classification, remains less explored. One potential approach is converting structural flow datasets into images, which has been proposed for tasks like malicious flow classification [150] and encryption flow classification [158]. This transformation allows dataset distillation to reduce resource consumption and improve the time efficiency in flow classification tasks.
  Furthermore, recent studies indicate that text-based network traffic flows can be represented as bit arrays, enabling high-accuracy malicious traffic classification [159,160]. This suggests that dataset distillation techniques, whether image-based or not, can be applied to non-image classification tasks as well. Additionally, the relationship between decision trees and neural networks has been explored, opening the possibility of using decision trees and decision tree-based algorithms for distilling datasets in non-image tasks.
  In cases where gradient descent methods are not applicable, generic search algorithms, such as grid search and genetic algorithms, can be used to optimize synthetic datasets for non-image classification models. This broadens the scope of dataset distillation, making it adaptable to various types of machine learning models beyond neural networks.
- Combining multiple data condensation techniques: Given the resource constraints, concept drifts, and catastrophic forgetting issues prevalent in current network traffic classification scenarios, it is essential to combine various data condensation techniques. Classic techniques like coreset selection and data compression can be integrated with advanced methods such as model and dataset distillation to enhance performance in resource-constrained environments.
  In knowledge distillation, it is crucial to compress the teacher model by eliminating redundancies before transferring knowledge to a student model. In dataset distillation, constructing a coreset from the original dataset plays a vital role in reducing dataset size and guiding the distillation of the synthetic dataset. Compression techniques can also be applied to real-world datasets to minimize redundancy and reduce training time in flow classification tasks.
  To address real-world time and resource limitations, a combination of multiple dataset condensation techniques is necessary. Given that network traffic is often encrypted and compressed for privacy and resource efficiency, investigating malicious flow models on encrypted and compressed network traffic has become a key area of research [143,144].
- Applying data condensation on the network edge: Network edge devices are typically characterized by resource constraints in terms of computation, storage, bandwidth, and power, making it challenging to deploy traffic classification applications effectively. Data condensation plays a critical role in enhancing the efficiency and feasibility of such applications on these devices. Given that these network devices are commonly used in environments like wireless sensor networks, mobile networks, and vehicular ad hoc networks, which have stringent performance requirements—such as low response times, high robustness, and minimal latency—deploying network traffic classification tasks becomes highly complex.
  Additionally, migrating from large models to smaller, more efficient ones without significant performance degradation, ensuring robust data privacy, maintaining low latency, and improving network interoperability all add layers of complexity to the deployment process. Techniques like data condensation on programmable network datapaths (e.g., P4) and offloading in SDN devices [161] can enhance network performance while enabling in-network intelligence, providing a pathway to address these deployment challenges.

## 7. Conclusions

This paper provides a systematic literature review of data and knowledge condensation methods aimed at reducing traffic datasets, simplifying traffic models, and transferring network knowledge for network traffic classification tasks in dynamic, resource-constrained environments. The review covers both classical techniques, such as coresets and compression, and advanced methods like knowledge distillation and dataset distillation. Each technique is introduced with its core concept and categorized based on data types, methods, and applications.

We highlight that the large size of network traffic data presents significant resource constraints, hindering effective classification at the network edge. Data condensation methods are crucial for overcoming these limitations. The review demonstrates that, while classical condensation techniques are simple and effective, they struggle to provide the desired accuracy in resource-constrained environments, especially when data or models require migration. Advanced techniques, by addressing issues like catastrophic forgetting in the face of concept drift, offer negligible or even improved accuracy, especially when knowledge needs to be transferred across models and datasets. However, these advanced methods are currently limited to neural network-based image classification tasks and do not yet accommodate text-based or tabular network traffic data.

We underscore the need for innovation in knowledge and dataset distillation techniques, which could be integrated into flow traffic classification using continual and federated learning approaches. This integration could overcome challenges in current traffic classification tasks within evolving network environments. Future research should focus on addressing issues such as class imbalance, non-neural network-based distillation, combining multiple condensation techniques, and applying data condensation at the network edge to tackle these challenges effectively.

## Figures and Tables

**Table 1:** The related surveys in current research.

Domain	Survey	Purpose and Contents
Coreset selection	[7]	Methods, application, open issues
	[8]	Methods targeting accurate coresets
	[9]	Build coresets for time series
Data compression	[10]	Approaches and open issues
	[11]	Compress images for wireless sensor networks
	[12]	Compress videos
	[13]	Compress time series
	[14]	Two-dimensional compression algorithms
	[15]	Three-dimensional compression algorithms
Knowledge distillation	[16]	Architectures, algorithms, applications
	[17]	Knowledge/architectures/algorithms/applications
	[18]	What/who/how to distill in graphs
	[19]	In deep learning
	[20]	Graph-based distillation approaches
	[21]	Distill diffusion models
	[22]	For computer vision
	[23]	For medical field
Dataset distillation	[24]	Applications
	[25]	Algorithms and performance
	[26]	Algorithms

**Table 2:** The categories of coresets.

Type	Subtype	Description
Data type	Weighted subset of input	Set of weighted input
	Weighted subset of input space	Set of data from the same ground set
	Sketch matrices	Set of linear combination of inputs
	Low-dimensional coresets	Data in low dimensional space
	Generic data structures	Hybrid of all above
Query set	Strong	Approximate any given query in the given query set
	Weak	Provide error guarantees for some not all queries
	Sparse	Providing error guarantees for the optimal query
Construction	Uniform sampling	Group inputs and take samples from each group
		Proportional to the size of the group
	Significance sampling	Take samples from a distribution approximated by a weighted
		Average of random draws from the distribution of inputs
	Grid sampling	Split input space into cells and take a sample from each cell
	Greedy construction	Repeatedly pick the next best point based on specific criterion

**Table 3:** The categories of compression.

Category	Subcategory	Description
Types of codes	Block–block	Input and compressed data has the same length
	Block–variable	Input has fixed length but compressed data has various length
	Variable–block	Input has various length but compressed data has fixed length
	Variable–variable	Both input and compressed data have various length
Types of data	Text	Reduce the body of texts and remove the redundancy
	Image	Reduce the size of image for transmission and storage
	Audio	Compress music and speech, decoder intensive
	vedio	Reduce the redundancy in both spatial and temporal dimensions
	Model	Compress the parameters of huge models in deep learning
Data quality	Lossy	Information loss after compression
	lossless	no information loss after compression
Coding scheme	Huffman coding	Determine minimum cost prefix-free codes
	Arithmetic coding	Store frequently used characters in fewer bits and vice versa
	Dictionary-based coding	Find patterns in input, code patterns based on a dictionary
	Burrows wheeler transform	Permutate input for easier compression
	Fractal compression	Use fractals to compress digital images
	Wavelet transform	Use wavelets to transform time-space input to time-frequency codes
	Quantization	Reduce the precision of input datatype for less computation cost

**Table 4:** The categories of knowledge distillation.

Category	Subcategory	Description
Knowledge	Response-based	Knowledge extracted from output layer
	Feature-based	Knowledge extracted from intermediate layers
	Relation-based	Relationships between layers of models
Schemes	Offline	Pre-trained teacher model guides the student model
	Online	Teacher and student models are jointly updated online
	Self	Same model used to train both teacher and student online
Algorithms	Adversarial	Use generated adversarial samples in training set
	Multi-teacher	Convert knowledge from multiple teachers
	Cross-modal	Transfer knowledge among multiple modalities
Applications	Visual recognition	Simply models, improve accuracy and efficiency
	Natural language processing	Reduce model size and improve time efficiency
	Speech recognition	Real-time systems in embedded platforms
	Others	Recommendation and systems protecting models being attacking

**Table 5:** The categories of dataset distillation.

Category	Subcategory	Description
Methods	Performance matching	Tune synthetic set to match the performance of models that are trained on the synthetic and original sets, respectively,
	Parameter matching	Tune synthetic set to match the parameters of models that are trained on the synthetic and original sets, respectively
	Distribution matching	Tune synthetic set so that the distribution of both synthetic and original can be matched
Applications	Continual learning	Generating a small synthetic set capturing the knowledge in the previous training set and add the synthetic set to the current training set
	Federated learning	Exchange the synthetic set distilled from the set of each client to reduce communication cost while maintaining data privacy
	Privacy and security	Use synthetic set distilled from the original set for privacy protection and avoid attacks
	Others	Used synthetics set in recommendation, text classifications, and medical systems to reduce the training set and protect the data privacy

## Data Availability

Not applicable.

## Abbreviations

ASCII	American Standard Code for Information Interchange
EBCDIC	Extended Binary Coded Decimal Interchange Code
GNN	Graph Neural Network
IoT	Internet of Things
VANET	Vehicular Ad Hoc Network
SDN	Software-Defined Networking
SQL	Structured Query Language
TCP	Transmission Control Protocol
UDP	User Datagram Protocol
URL	Uniform Resource Locator
XML	Extensible Markup Language
